# Physical activity reduces anxiety and regulates brain fatty acid synthesis

**DOI:** 10.1186/s13041-020-00592-7

**Published:** 2020-04-17

**Authors:** Arkadiusz Liśkiewicz, Marta Przybyła, Anna Wojakowska, Łukasz Marczak, Katarzyna Bogus, Marta Nowacka-Chmielewska, Daniela Liśkiewicz, Andrzej Małecki, Jarosław Barski, Joanna Lewin-Kowalik, Michal Toborek

**Affiliations:** 1 Laboratory of Molecular Biology, Institute of Physiotherapy and Health Sciences, The Jerzy Kukuczka Academy of Physical Education, Mikołowska 72a, 40-065 Katowice, Poland; 2 Department of Physiology, Faculty of Medical Sciences in Katowice, Medical University of Silesia, Medyków 18, Katowice, 40-752 Poland; 3 Department for Experimental Medicine, Faculty of Medical Sciences in Katowice, Medical University of Silesia, Medyków 4, Katowice, 40-752 Poland; 4 Institute of Bioorganic Chemistry, Polish Academy of Sciences, 61-704 Poznan, Poland; 5 Department of Histology, Faculty of Medical Sciences in Katowice, Medical University of Silesia, Medyków 18, 40-752 Katowice, Poland; 6 Department of Biochemistry and Molecular Biology, University of Miami School of Medicine, 1011 NW 15th Street, Miami, FL 33136 USA

**Keywords:** Physical activity, Neurobehavior, Metabolome, Neurogenesis

## Abstract

Physical activity impacts brain functions, but the direct mechanisms of this effect are not fully recognized or understood. Among multidimensional changes induced by physical activity, brain fatty acids (FA) appear to play an important role; however, the knowledge in this area is particularly scarce. Here we performed global metabolomics profiling of the hippocampus and the frontal cortex (FC) in a model of voluntary running in mice. Examined brain structures responded differentially to physical activity. Specifically, the markers of the tricarboxylic acid (TCA) cycle were downregulated in the FC, whereas glycolysis was enhanced in the hippocampus. Physical activity stimulated production of myristic, palmitic and stearic FA; i.e., the primary end products of de novo lipogenesis in the brain, which was accompanied by increased expression of hippocampal fatty acid synthase (FASN), suggesting stimulation of lipid synthesis. The changes in the brain fatty acid profile were associated with reduced anxiety level in the running mice. Overall, the study examines exercise-related metabolic changes in the brain and links them to behavioral outcomes.

## Introduction

Physical activity has been associated with a plethora of functional, cellular, and molecular alterations within the brain. It is widely known that exercise improves mood and cognition, accelerating hippocampal neurogenesis in humans and in animal models. Voluntary wheel running correlates with an increase in the gene expression of neurotrophins and other growth factors [1, 2], markers of synaptic plasticity, and downregulation of inflammatory factors [3], enhancing cognitive functions [4, 5]. Beside its pro-cognitive role, exercise improves mood and libido but little has been known about why it has such a profound effect on the brain [6]. Exercise, among other behavioral regulations, appears to reduce anxiety level (anxiolytic effect) serving as a potential additional therapy in behavioral disorders [7]. Nevertheless, these results remain controversial. While decreased levels of anxiety-related behaviors have been observed after voluntary wheel running [8, 9], other studies have reported increased anxiety-like behaviors following exercise [10] or have failed to find any effect of exercise on anxiety levels [11].

As a multifactorial determinant exercise impacts overall tissue biology, and modification of metabolomic composition may explain er central consequences of physical activity [12]. The brain metabolome analysis provides a large-scale quantitative and qualitative profiling of the metabolites that are the essential components of the brain functioning, offering a key opportunity to advance in neuroscience [13]. In the context of the neuroscientific research, several groups of metabolites, such as energetic substrates, neurotransmitters, neurochemicals, and structural lipids can be distinguished [14]. The knowledge about functional outcomes of affected metabolic composition of the brain is extensive in terms of neurotransmitters but scarce for the other aforementioned groups of metabolites. Remarkably, the role of brain lipid composition has been considered to influence the brain homeostasis [15–17]. However, it is not validated how the brain metabolites are affected by exercise.

The frontal cortex (FC) and the hippocampus are well-described brain structures involved not only in memory and cognitive function, but also in mood regulation, such as depression and anxiety [18]. These structures are also of particular importance to recent animal studies related to the beneficial effects of physical activity on the brain. Therefore, the goal of the present study was to apply global metabolic profiling in cortical and hippocampal samples of exercised mice and inactive controls, and to link the obtained results to functional outcomes. This article describes the changes in the composition of the hippocampal and cortical metabolites, opening the discussion about a role of fatty acids (FA) in anxiolytic effect caused by physical activity.

## Materials and methods

### Animals and experimental design

All animals were provided by the Animal House of the Department for Experimental Medicine, Medical University of Silesia, Katowice, Poland, and were treated in accordance to the Directive 2010/63/EU for animal experiments using the protocols approved and monitored by the Local Ethics Committee for Animal Experimentation in Katowice. All animals were housed in separate cages in a 12:12 light-dark (LD) cycle environment, with light presented at 7.00 a.m. All experiments were performed during the light phase. The water and standard rodent chow were provided ad libitum.

**Fig. 1:**
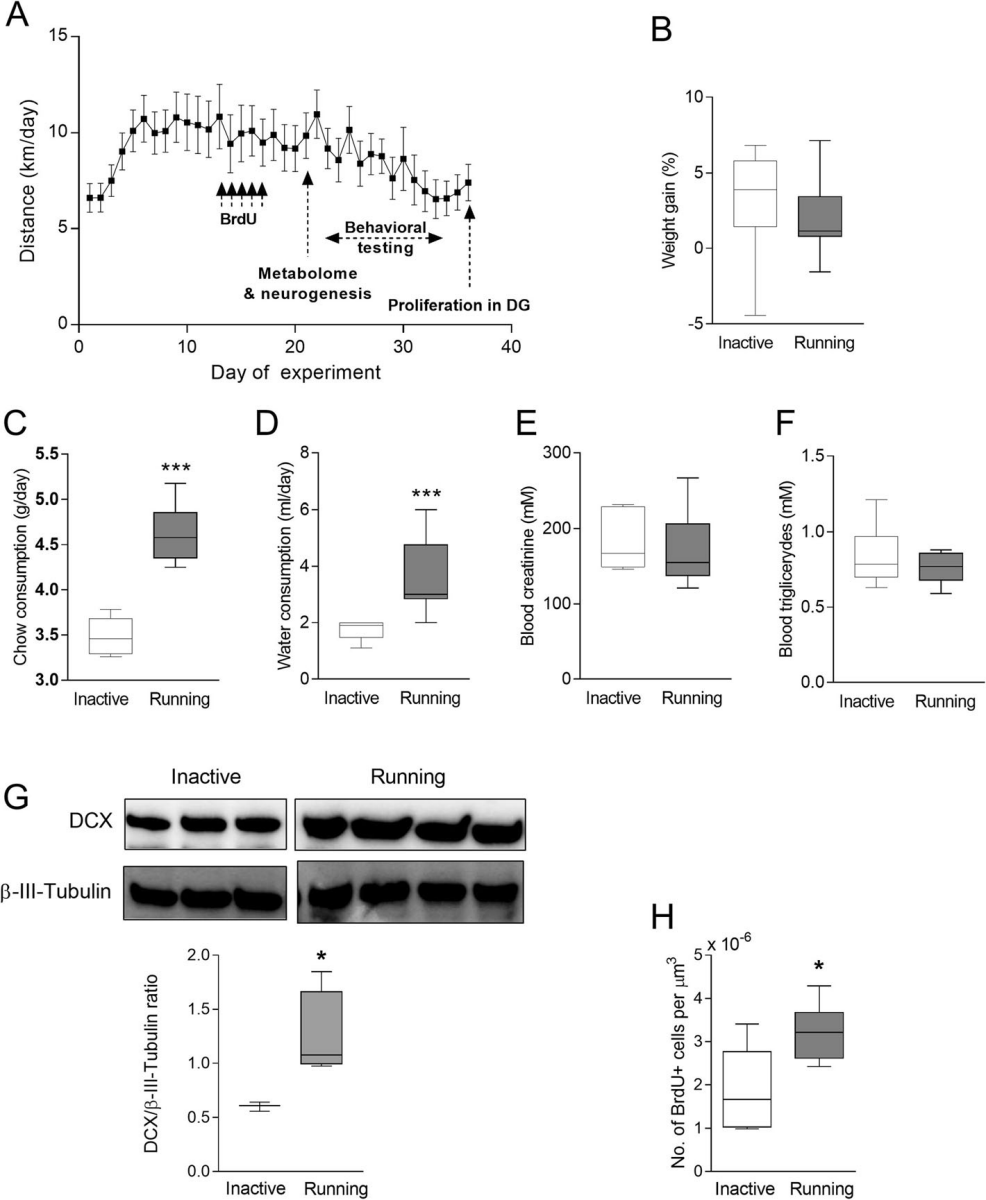
Characterization of the running mouse model and experimental timeline. (**a**) Distance traveled by mice provided with running wheels for up to 36 days. Data are mean +/- S.D; n=14. The graph includes also the timeline of experiments. Vertical arrows indicate BrdU injections, evaluation of metabolome and neurogenesis, behavioral testing, and cell proliferation in the dentate gyrus (DG). Weight gain (**b**), food consumption (**c**), water intake (**d**), blood creatinine (**e**) and triglyceride levels (**f**) determined during the entire running and/or inactive period (n=6-12). (**g**) Doublecortin (DCX, a neuronal differentiation marker) protein expression in the hippocampus of the running and inactive mice as determined by immunoblotting. Upper graph, representative images, lower graph, quantitative data normalized to β-III-tubulin levels (a housekeeping protein). (**h**) BrdU-positive cells in the hippocampus of the running and inactive mice. (**b-h**) graphs represent median with min. - max.; *p < 0.05, ***p < 0.001.

A cohort of 13-week-old C57BL/6NCrL male mice was divided into two groups: (i) running mice with access to the running wheels (Coulbourn Instruments, PA, US) and (ii) inactive (sedentary) mice that were housed in the same manner as the running mice; however, the running wheels were blocked as described earlier [19–21]. This nomenclature was based on the recommendations by the Sedentary Behavior Research Network [22]. The distance run in the wheels was recorded individually for all mice using Clocklab software (Actimetrics, IL, US). The experimental design is reflected in Fig. 1a. Separate cohorts of running and inactive mice were used to evaluate a) metabolome profile, b) neurogenesis, c) behavioral modification, and d) cell proliferation in the dentate gyrus of the hippocampus. The tissues for analyses were collected during the day at the resting phase (i.e., not at acute phase of the running) to capture the longterm effects of physical activity on the brain.

### Metabolomic profiling

#### Sample preparation and derivatization

Following 21 days of running, seven animals from the running and inactive groups were sacrificed by decapitation. The FC and the hippocampi were quickly dissected, immediately put on dry ice, and deep frozen (− 80 **°**C) until the metabolome profiling. Deep-frozen samples were then homogenized in a ball mill to a fine powder and 50 mg of each preparation was lysed in 200 μL of 1 M triethylammonium acetate buffer containing 0.1% SDS. Four volumes of cold methanol were added to obtained lysates and samples were kept in − 20 °C for 30 min. Next, samples were centrifuged at 11,000×g for 10 min and supernatants were dried in a vacuum centrifuge. After desiccation, each sample was supplemented with 50 μL methoxyamine hydrochloride (20 mg/mL in dry pyridine) and vortex-mixed in a thermomixer for 1.5 h at 37 °C; afterwards they were centrifuged for 10s. Following centrifugation, 80 μL of N-methyl-N-(trimethylsilyl)trifluoroacetamide (MSTFA) was added to each sample, vortex-mixed again in a thermomixer for 30 min at 37 °C, and centrifuged at 11,000×g for 10 min. Then, 100 μL of each sample was transferred to a conical glass vial prior the GC separation.

#### Gas chromatography coupled with mass spectrometry (GC-MS) and data analysis

Metabolites were identified and relatively quantified by GC-MS (TRACE 1310 GC oven with TSQ8000 triplequad MS from Thermo Scientific, USA), using a DB-5MS column (30 m × 0.25 mm × 0.25 μm, J&W Scientific, Agilent Technologies, Palo Alto, CA, USA). Gradient: 70 °C for 2 min, followed by 10 °C/min up to 300 °C (10 min). Injector 250 °C, interface 250 °C, source 250 °C, m/z range: 50–850, EI+, electron energy 70 eV.

Obtained RAW files were converted to CDF format and analyzed in LECO ChromaTOF software for automatic peak deconvolution, compound identification and sample alignment. Tabulated data were further used for comparative analysis which was done in Perseus software. For hierarchical clustering purposes data were normalized using Z-score algorithm. Clustering was performed on both rows (proteins) and columns (samples) by calculating Euclidean distances, and the obtained data were presented as heat maps.

### Immunoblotting

A separate cohort of mice running for 21 days and inactive controls was decapitated for the brain collection to assess neurogenesis by evaluation of the formation of immature neurons that originate from neural progenitor cells in the dentate gyrus of the hippocampus. Doublecortin (DCX) is a microtubule-associated protein expressed in immature, i.e., migrating and differentiating neurons; therefore, it was determined as a marker of neurogenesis. Immunoblotting was performed as described previously [23]. Briefly, frozen brain samples were homogenized in RIPA buffer (Millipore, St. Charles, MO, USA) supplemented with protease inhibitor cocktail tablets (cOmplete, Roche, Germany). Homogenates were centrifuged at 14,000×*g* for 15 min and the supernatants were used for immunoblotting. Protein concentrations were determined using RotiQuant Protein Assay Kit (Carl Roth, Germany). Samples were separated on 4–15% SDS-PAGE and transferred onto PVDF membranes (Bio-Rad Laboratories, CA, USA). Membranes were blocked for 1 h at room temperature in Casein Blocking Buffer (Sigma-Aldrich) and incubated overnight at 4 °C with rabbit anti-DCX (1:1000, ab18723, Abcam, UK) or with anti-βIII-Tubulin (a housekeeping protein) (1:1000, ab78078, Abcam, UK). Individual immunoblots were visualized by a Clarity ECL detection kit (Amersham Biosciences, Piscataway, NJ), DCX expression was quantified by Image Lab 6.0.1 software (Bio-Rad Laboratories, CA, USA), normalized to βIII-tubulin levels, and used for statistical analysis.

Similarly, the cortical and hippocampal samples were used to assess expression fatty acid synthase (FASN; 1:1000, ab128870, Abcam, UK) and myelin basic protein (MBP; 1:1000, ab40390, Abcam), the key enzyme for de novo FA biosynthesis and a marker of myelin, respectively. FASN and MBP expression was normalized to total protein (Stain free technique, Bio-Rad) and not to housekeeping protein, because we observed that β-actin expression was lower in all cortical samples as compared to the hippocampus (data not shown). The Stain-free imaging technology utilizes a proprietary polyacrylamide gel chemistry to induce protein fluorescence directly on the gel with a short photoactivation, and allowing the immediate visualization of proteins at any point during electrophoresis and blotting.

### Behavioral tests

A separate cohort of the running and inactive mice was subjected to behavioral testing to correlate anxiolytic behavior with metabolome changes.

#### Open field (OF) anxiety test

The OF test, which measures locomotor and exploratory activity, was performed by placing the mice for 10 min in a transparent square cage (40 × 40 cm) located in a new, unknown room. Animal behavior was recorded by infrared detectors (TruScan, Coulbourn Instruments, PA, USA), and the movements, total distance, and velocity were calculated. Center area was defined as more than three photobeams (3 cm) from the side walls.

#### The elevated plus maze (EPM) anxiety test

The animals were subjected to a plus shape area with two open and two closed arms (30 × 5 cm), which was raised 40 cm above the floor. At the start of each test, mice were individually placed on the central platform, and their behavior was monitored by the video camera for 5 min. The animal’s movement was analyzed by EthoVision XT 10 software (Noldus Information Technology, The Netherlands). The time spent in each arm (open and closed) and the time of head-dipping were calculated.

#### The dark/light (D/L) anxiety test

The D/L test is a modification of the OF test that is used for further assessment of anxiety-like behavior. The apparatus consists of two equal square compartments, where one compartment is illuminated and the other one is covered with a black box. Both compartments are connected with an opening enabling free communication between both parts. The mice were placed in the illuminated compartment, and the test lasted 10 min. The time spent in each compartment was scored for each animal.

### Cell proliferation in the hippocampal dentate gyrus

Following 2 weeks of running, six mice from each group were injected intraperitoneally once daily for 5 days with bromodeoxyuridine (BrdU) at the dose of 150 μg/g in a saline solution, labeling newly proliferating cells. The mice continued to run for the additional 20 days, allowing us to assess a long-term impact of physical activity on cell proliferation in the dentate gyrus of the hippocampus. After the animals were euthanized, the brains were collected and immersed in a 10% formalin solution (pH 7.4) in TBS for 24 h. The formalin fixed brains were sectioned coronally (25 μm thick) and then stored in 50% glycerol in TBS at − 20 °C. Proliferating cells in the dentate gyrus were detected by immunostaining with anti-BrdU antibody (1:300, ab1893, Abcam) conjugated with Alexa Fluor 588 secondary antibody (1:500, ab150177, Abcam). Afterwards, slices were washed and mounted onto glass microscope slides with Glycerol Mounting Medium with DAPI and DABCO™ (ab188804, Abcam). Images of the dentate gyri of the left and right hippocampi were taken and z-stacked using a 20x objective and a confocal microscopy. The BrdU-positive cells were then counted manually and expressed per volume (μm^3^). The results from the left and right hippocampi were averaged and multiplied by two to reflect the total number of proliferating cells in both hippocampi.

### Statistical analysis

Prism 8.0 (GraphPad Software, CA, US) was used for statistical analyses and figure generation. Student’s t-test or U Mann-Whitney test were used for data analysis. Wilcoxon matched-pairs signed rank test was used for paired data. One-way-ANOVA with Fisher post hoc was used to compare immunoblotting data for FASN expression. Data on the graphs was expressed as median +/− min. to max, unless otherwise stated. In all analyses, the *p* value less than 0.05 was considered to be statistically significant.

## Results

### Characterization of the running mouse model

We have used a wheel running mouse model to evaluate the influence of spontaneous and voluntary exercise on physiological aspects of the brain. Within the first 5 days after getting access to the running wheels, mice adjusted to this new task and ran with increasing intensity, reaching the stable level of about 8–10 km per day from day 6 (Fig. 1a). Systemic adaptation of mice to exercise requires a 3 week running period [24]; therefore, our measurements were performed after 21 days of running. The animals were active almost exclusively during the dark phase of the 12:12 daily cycle (Supplementary Figure S1). As shown in Fig. 1a, the mice decreased spontaneous wheel-running starting at day 22; however, they still run a total distance in the range from ∼6 to 10 km, which has been reported as typical for C57BL/6 J mice [24]. This drop of the running intensity was not observed in the mice predisposed to BrdU evaluation (i.e. the mice that were not been subjected to behavioral testing; data not shown), suggesting that the behavioral evaluation during the day affected the running activity at nights. To match the description with Fig. 1 please make the order of the sentences as follow: The running mice gained approximately the same weight as the inactive controls (Fig. 1b), despite increased food intake (*p* < 0.0001; Fig. 1c) which was accompanied by higher water consumption (*p* < 0.001; Fig. 1d). No features of fatigue or changes in cognitive abilities (data not shown) were noted in the running mice, the findings that were consistent with the literature [25, 26]. We also did not detect any changes in plasma creatinine level, the marker of muscle breakdown, as the result of wheel running (Fig. 1e). All these observations are consistent with the fact that physical activity was fully voluntary and spontaneous. We also measured the level of triglycerides in the blood, and no differences were noted in the running group as compared to inactive mice (Fig. 1f).

To further validate the model, we evaluated neurogenesis which typically increases in exercised animals [27]. Specifically, we examined the levels of doublecortin (DCX), a marker of pro-neuronal differentiation, in the hippocampal dentate gyrus. DCX expression was significantly higher in the running mice than in controls (Fig. 1g, *p* = 0.029), indicating enhanced differentiation into the neuronal lineage as a result of increased physical activity.

We also evaluated the number of proliferating cells in the dentate gyrus. Following 14 days of voluntary wheel running, mice received five injections with BrdU to label proliferating cells. To study a long-term effect of physical exercise, mice were allowed to maintain physical activity for the additional 20 days. The length of exercise was chosen based on the fact that immature newborn granule cells enter an integrated stage as early as 2 weeks after being born, and we extended this time frame by the factor of 2 to more precisely capture cell proliferation. Because of the high density of neuronal progenitor cells in the dentate gyrus, the method primarily reflects proliferation of this cell type. A significantly higher number of BrdU-positive cells was detected in the dentate gyrus from the running mice (Fig. 1h; *p* = 0.029) as compared to inactive controls, suggesting enhanced cell proliferation and/or survival.

### Bioenergetics metabolites are distinctly expressed in the FC and hippocampus in response to exercise

**Fig. 2:**
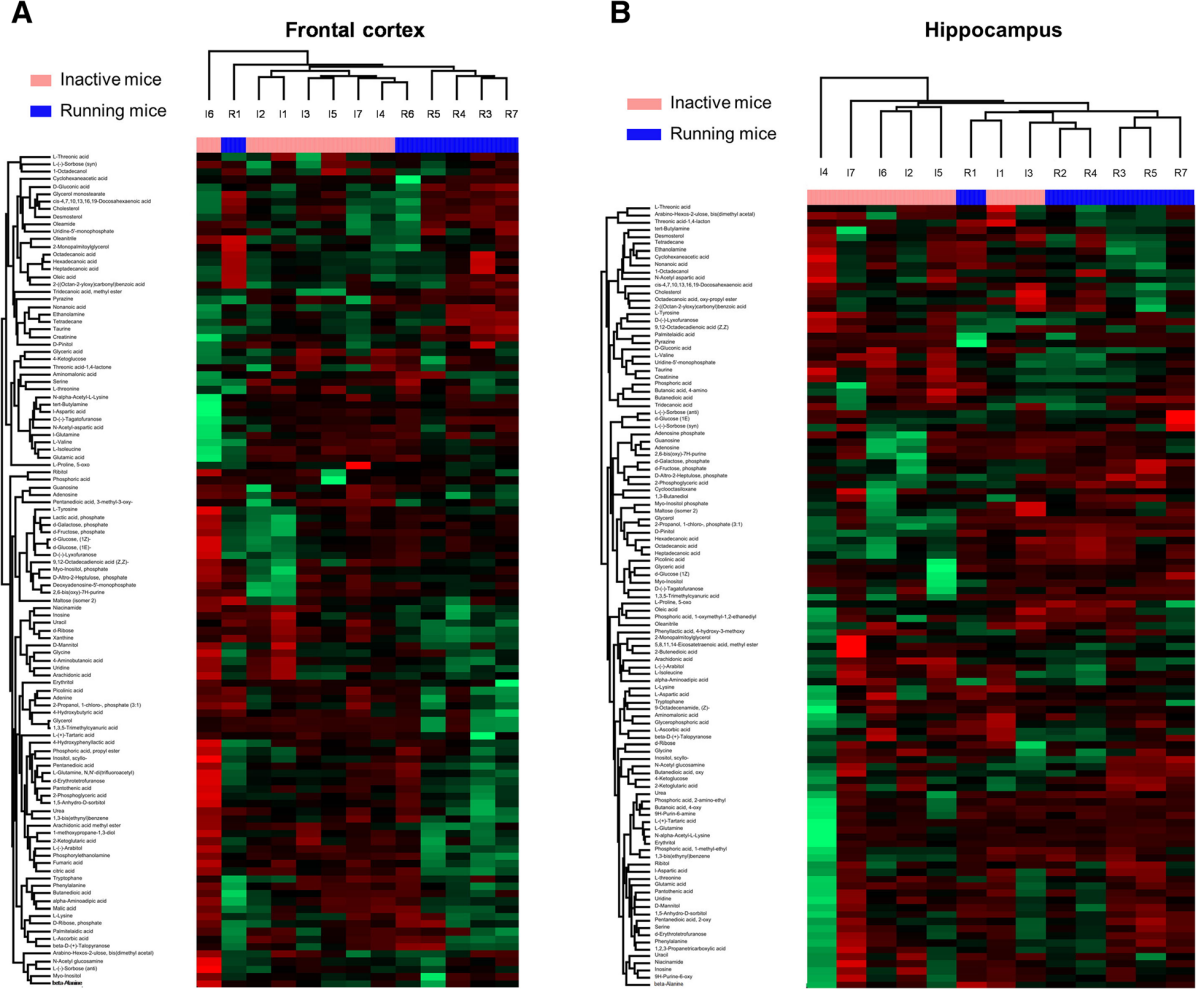
A heat map of identified compounds in metabolomic profiling of (**a**) the frontal cortex and (**b**) the hippocampus. Samples were collected and analyzed from the inactive (I 1–7) or running (R 1–7) mice; *n* = 6–7

We analyzed the impact of physical activity on the metabolomic profile in two brain structures, namely in the hippocampus and the FC. Overall, 111 metabolites were identified (Fig. 2a-b), with 16 significantly changed in the hippocampus, and 15 significantly altered in the FC. All compounds that differed significantly between the running and inactive groups are presented in Supplementary Table S1.

**Fig. 3:**
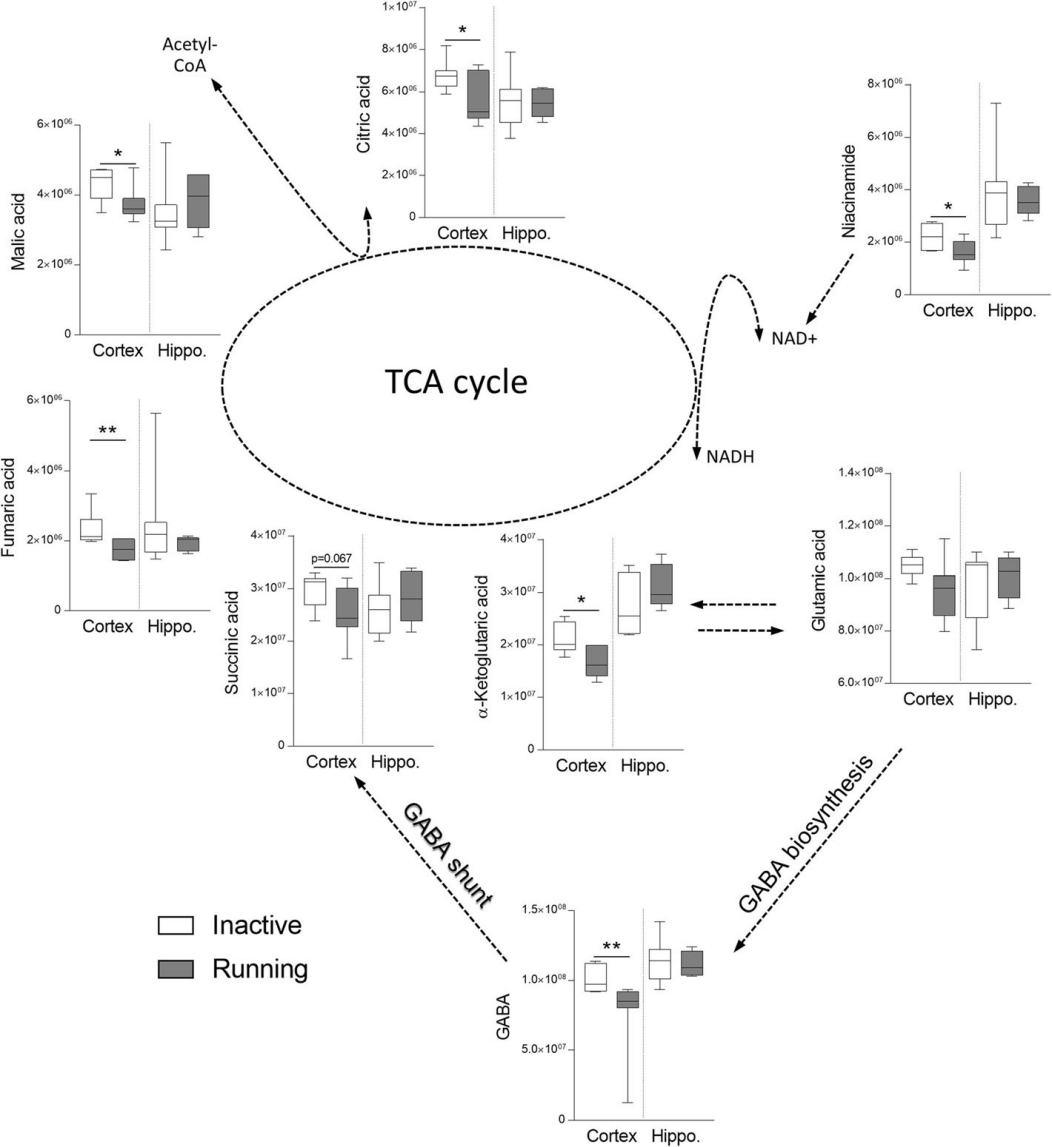
Visual representation of the changes in the frontal cortex and the hippocampal metabolites related to the TCA cycle. Decreased concentrations of citric, succinic, fumaric, and malic acids, with concomitant decrease of niacinamide suggest downregulation of the TCA cycle in the frontal cortex of the running mice. Glutamic and alpha-ketoglutaric acids participate in endogenous GABA production, which is reduced in line with the TCA turnover. In the hippocampus, the metabolites did not differ significantly between the groups. The inactive and running groups were compared separately for the frontal cortex (Cortex) and the hippocampi (Hippo) as marked by the dotted line. Median with min. - max. *p < 0.05, **p < 0.01; *n* = 6–7

The analysis revealed distinct metabolic responses to exercise in the hippocampus and the FC. Interestingly, the levels of the TCA cycle intermediates were not altered in the hippocampus, while a significant decrease was noted in the FC in the running mice as compared to their inactive controls. The levels of citric acid (*p* = 0.034), alpha-ketoglutaric acid (*p* = 0.048), fumaric acid (*p* = 0.008), and malic acid (*p* = 0.045) were all reduced in the FC of the running mice (Fig. 3). The concentration of niacinamide, the NAD+ donor in the TCA cycle was significantly lowered (*p* = 0.031) in the running mice. The TCA results are different from those obtained in a rat model of forced and exhaustive exercise, likely because samples in our study were collected during a resting phase [28]. Our results based on a prolonged voluntary exercise indicated a decrease in the TCA metabolites and glycerol (an energetic substrate utilized by neurons [29], *p* = 0.038) in the FC of the running mice, with a simultaneous increase in glycolysis metabolites in the hippocampus (Table 1). An increase in the glycolytic metabolites, such as 3-phosphoglycerate (*p* = 0.0133) and fructose 6-phosphate (*p* = 0.026) in the hippocampi of the running mice is consistent with previous reports on upregulation of the hippocampal enzymes involved in glucose utilization and metabolism in rodent models of physical activity [30–32]. Observed in our study was a decrease in the level of cortical glycerol (*p* = 0.038), an energetic substrate utilized by neurons [29], may contribute to the insufficient TCA turnover (Table 1). A decrease in cortical GABA level (*p* = 0.0023, Fig. 3) may also be related to the inefficient TCA cycle, because this neurotransmitter can be synthesized by glutamate/alpha-ketoglutarate/GABA pathway [33]. The median concentration of glutamic acid, the crucial neurotransmitter and substrate for the GABA synthesis in the pathway mediated by alpha-ketoglutaric acid [34, 35] showed a tendency to be decreased in the FC of the running mice (Fig. 3). Despite the decreased cortical GABA level, the GABA/glutamate ratio was not changed (0.92 [0.83–1.16] vs 0.89 [0.11–1.15] for the running vs inactive mice, respectively). The right balance of GABA/glutamate is essential for normal brain functioning [36]; thus, the lowered cortical GABA probably does not affect behavioral phenotype in our running mice.

**Table 1:** Additional clusters of brain bioenergetic metabolites in response to exercise in the hippocampus and the frontal cortex of running mice presented as % of control (*n* = 6–7)

No.	Compound name	Hippocampus		Cortex		Function
		Runners (%)	***P*** value	Runners (%)	***P*** value	
**Glycolysis**						
1.	Fructose 6-phosphate	↑ 163	< 0.05	90.8	ns	Glucose-6-phosphate is converted to fructose-6-phosphate in the second step of the glycolytic pathway.
2.	3-Phosphoglycerate	↑ 176.2	< 0.05	85.11	ns	3-carbon molecule that is a metabolic intermediate in glycolysis.
**Energetic substrate**						
3.	Glycerol	18,838	ns	↓ 68.9	< 0.05	Marker of triglycerides breakdown. Alternative TCA substrate.
**Amino acids and its metabolites**						
4.	Phenylalanine	99.5	ns	↓ 83.1	< 0.05	Essential amino acid and the precursor of the amino acid tyrosine. Like tyrosine, phenylalanine is also a precursor for catecholamines including tyramine, dopamine, epinephrine, and norepinephrine.
5.	Valine	↓ 84.9	< 0.05	98.7	ns	Essential branched-chain amino acid.
6	Picolinic acid	↑ 237	< 0.05	80	ns	Metabolite of the tryptophan catabolism via kynurenine pathway.
**Marker of food consumption**						
7.	D-Pinitol	↑ 431	< 0.001	132.6	ns	Biomarker of the consumption of soy beans and other soy products.

Amino acids can be converted to metabolic intermediates and utilized as the TCA substrates. The level of phenylalanine was decreased (*p* = 0.025) in the FC and the level of L-valine dropped (*p* = 0.022) in the hippocampi of the running mice. D-Pinitol, a marker of consumption of soy products [37], was elevated in the hippocampi of the running mice compared to the controls (*p* < 0.0001), suggesting a possible accumulation of this metabolite as the result of increased consumption of standard rodent’s chow that contains 14% of Hi-Pro soybean meal. This suggests another possible mechanism through which voluntary exercise may contribute to the modulation of the metabolome, namely, by increasing consumption of food and deposition of foodborne compounds in the brain.

### Physical activity alters FA profile in the brain

**Fig. 4:**
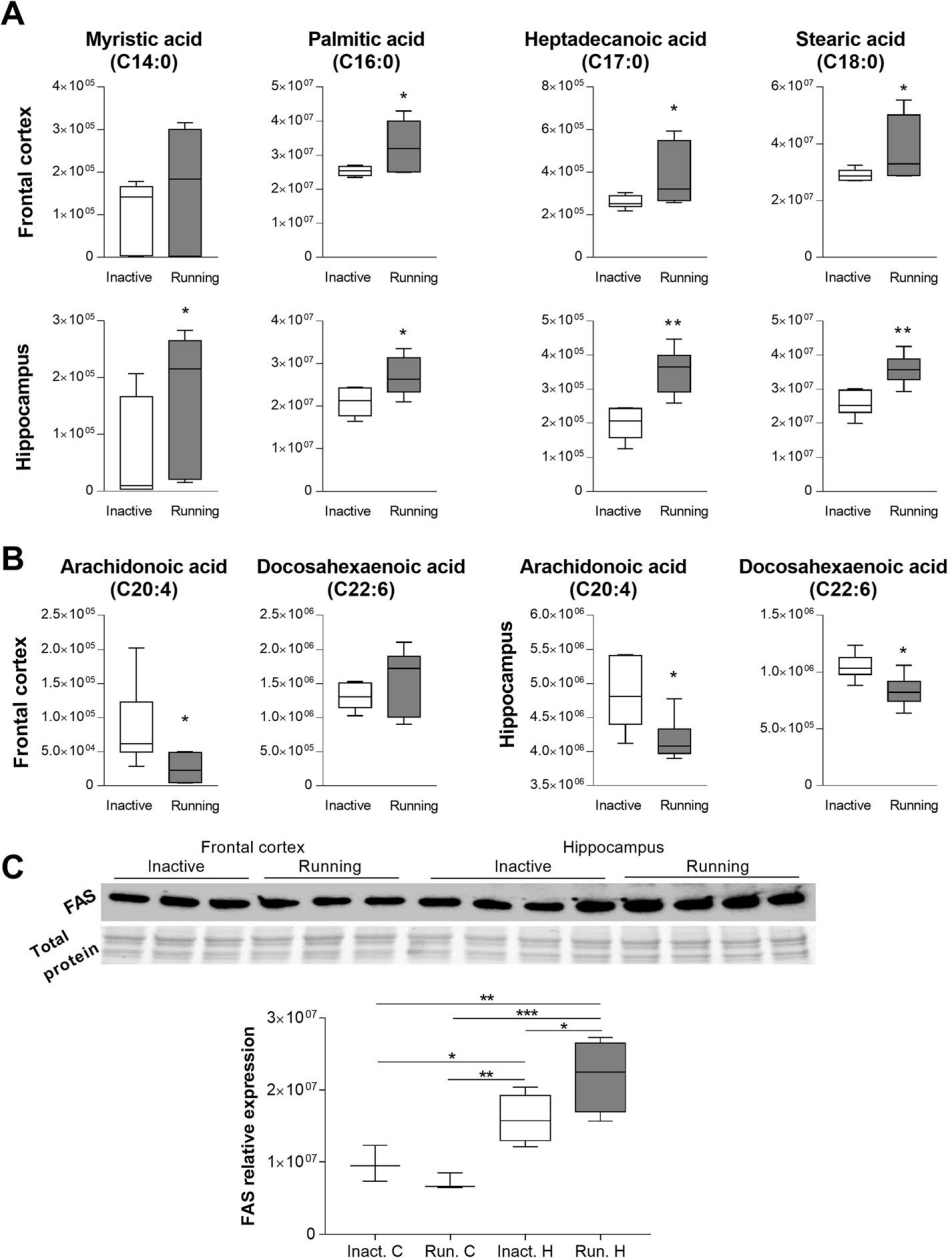
Impact of exercise on brain fatty acid profile. Frontal cortex and hippocampal samples were assayed for the profile of saturated (**a**) and unsaturated (**b**) fatty acids by metabolomics. **c** Immunoblotting was performed to assay the expression of the fatty acid synthase (FASN) protein in the running and inactive mice. Exercise upregulated FASN expression in the hippocampus but not in the frontal cortex. Upper graph, representative images, lower graph, quantitative data normalized to total protein level. Median with min. - max. *p < 0.05, **p < 0.01, *p < 0.05, **p < 0.01; *n* = 6–7

Analysis of the content of the hippocampal and FC samples showed remarkable differences in the FA profile due to physical activity (Fig. 4). Saturated FA (Fig. 4a), such as heptadecanoic (17:0), stearic (18:0), and palmitic acids (16:0) were increased in the running mice as compared to inactive controls in both the hippocampi and the FC. Myristic acid (14:0), another common saturated FA, was increased in the hippocampal samples, and showed a high tendency to be elevated in the FC. In contrast, unsaturated FA were generally decreased in the running mice (Fig. 4b). Specifically, the levels of arachidonic acid decreased in both examined brain regions of the running mice (*p* = 0.032 for FC and *p* = 0.021 for the hippocampus). In addition, the concentration of docosahexaenoic acid was lowered in the hippocampal samples (*p* = 0.015) but it tended to be increased in the FC (Fig. 4b).

Because palmitate, and to lesser extent, myristate and stearate are the primary end products of de novo lipogenesis [38], we hypothesized that this process may be boosted in the brain of the running mice. Therefore, we assessed the expression of FAS, which utilizes acetyl CoA and malonyl CoA to elongate FAs by two carbons catalyzing the biosynthesis of palmitate [39]. We observed that the levels of this maker of lipogenesis were elevated in the hippocampi but not in the FC of the running mice (Fig. 3c; ANOVA: F = 12.43, *p* = 0.001; results of post hoc testing are marked on the graph).

We also considered that the observed changes in FA composition could affect lipid enriched myelin sheath as a consequence of physical activity. To explore this possibility, we measured the expression of myelin basic protein (MBP), a marker of myelin, by immunoblotting. No changes were observed on the impact of exercise on the cortical and hippocampal level of this protein as compared to inactive controls (data not shown), suggesting that myelin levels were not changed in the running mice.

### Cortical and hippocampal metabolites correlate with activity level

We next evaluated a possible correlation between alterations of brain metabolites and mouse activity levels. Based on Pearson’s correlation, and we identified several metabolites which concentrations was positively or negative correlated with the level of activity calculated as the average daily distance run by an animal (Table 2).

**Table 2:** Results of Pearson’s correlation between the activity level and concentration of metabolites in the hippocampus or frontal cortex of the running mice

No.	Compound name	Pearson r value	***P*** value
**Hippocampus**			
1.	Galactose-6-phosphate	0.88	< 0.05
2.	Tyrosine	0.81	< 0.05
3.	Threonine	0.81	< 0.05
4.	Phenylalanine	0.81	< 0.05
**Cortex**			
5.	Citric acid	−0.78	< 0.05
6.	L-Arabitol	−0.98	< 0.0001
7.	L-Aspartic acid	−0.79	< 0.05
8.	Phosphoethanolamine	−0.82	< 0.05

The brain concentration of eight metabolites closely correlated with the activity level of the mice. In the hippocampus, tyrosine (*p* = 0.049), threonine (*p* = 0.048), phenylalanine (*p* = 0.048) and galactose-6-phosphate (*p* = 0.02) showed positive correlation with the distance run by mice. Phenylalanine, as a tyrosine precursor, is a substrate for tyrosine hydroxylase in the rate-limiting step in catecholamine synthesis [40]. Threonine regulates mTOR signaling [41], which is stimulated in the hippocampus and other brain structures of exercised rodents [42]. Galactose-6-phosphate is in constant equilibrium with fructose-6-phosphate, and the positive correlation between these two compounds was significant in the hippocampi of the running mice (*r* = 0.82, *p* < 0.05; data not shown). Among these metabolites, galactose-6-phosphate levels were increased in the running mice by 192.4% (*p* = 0.013) as compared to controls (Supplementary Table 1). In the FC, citric acid (*p* = 0.038), L-arabitol (*p* = 0.00014), aspartic acid (*p* = 0.035) and phosphoethanolamine (*p* = 0.022) negatively correlated with running activity level. Relatively to the inactive control mice, the cortical levels of citric acid, phosphoethanoloamine, and L-arabitol were 82% (*p* = 0.04), 76% (*p* = 0.04), and 68% (*p* = 0.0023), respectively. Cortical fumaric acid showed a tendency for negative correlation with activity level; however, the changes were not significant (*r* = − 0.71, *p* = 0.07). A very strong negative correlation (*r* = − 0.98, *p* < 0.0001) was observed for arabitol, a sugar alcohol, that can be found in most biofluids, although its role in living organisms is unclear. Information about its role in the brain is scare; however, in the light of the present findings, its functions should be reconsidered.

### Physical activity enhances anxiolytic behavior

**Fig. 5:**
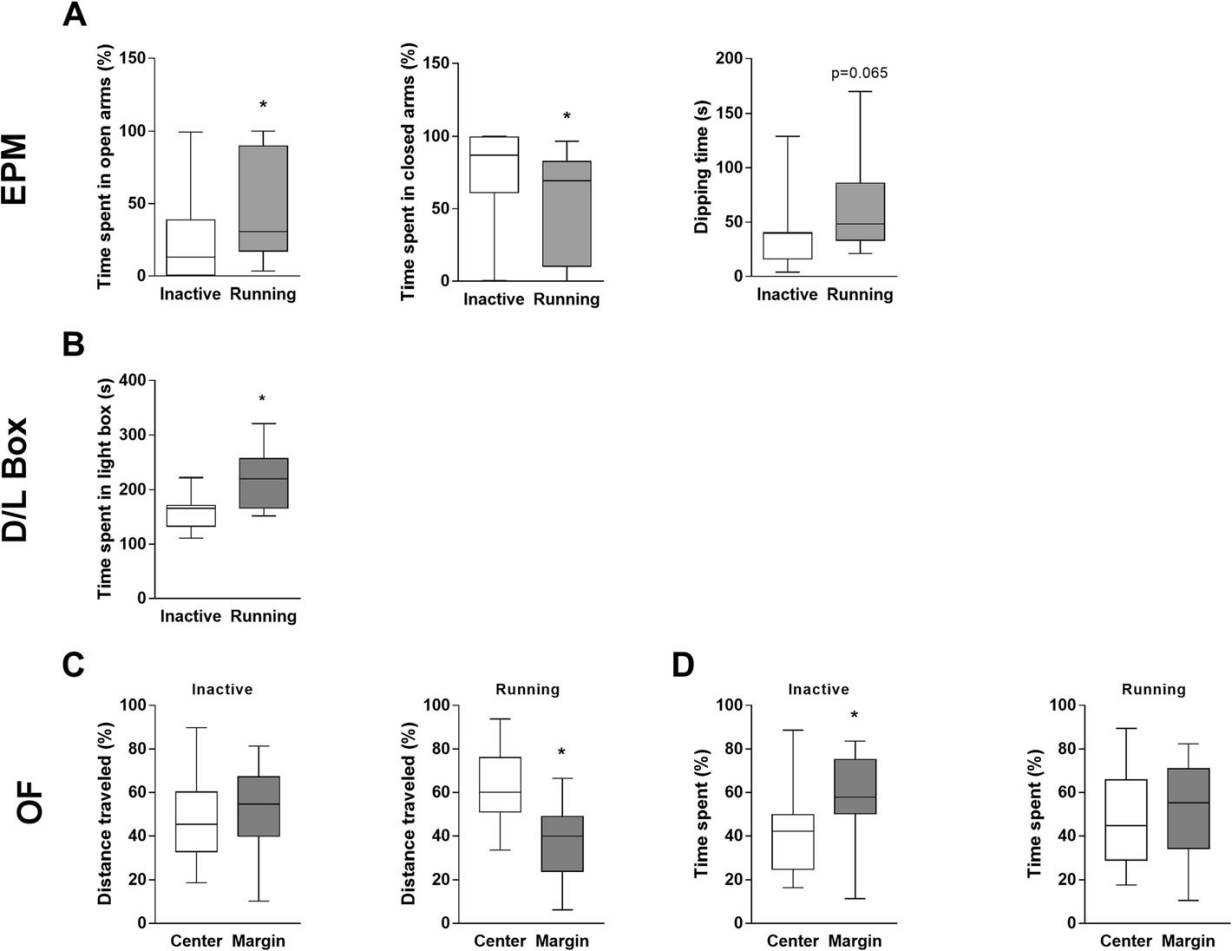
Impact of exercise on anxiolytic behavior. **a** Anxiolytic behavior as evaluated by the elevated plus maze (EPM) test. Analyses include time spent in the open arm (left), closed arm (middle), and the number of head dipping (right). **b** Anxiolytic behavior as evaluated by the dark/light (D/L) box test. The graph indicates time spent in the light box. **c-d** Anxiolytic behavior as evaluated by the Open Field (OF) test. The analyses include distance traveled (**c**) and time spent (**d**) in the center and margins of the cage by the inactive (left panels) and running (right panels) mice. Median with min. - max.; *n* = 15–16; *p < 0.05

Accumulation of even-chain saturated brain FA affect anxiety responses [43]; therefore, we also performed several tests focusing on anxiety evaluation in the running and inactive mice. In the EPM test, the running mice spent significantly more time in the open arms of the EPM area (*p* = 0.024, Fig. 5a, left panel) and less in the closed arms (*p* = 0.024, Fig. 5a, middle panel) than inactive animals, indicating enhanced anxiolytic responses. In addition, the cumulative time of nose exposing beyond the area (so called, dipping time) showed tendency to increase in the running mice (*p* = 0.065, Fig. 5a, right panel).

In the D/L test, the running mice spent significantly more time in the light area than the inactive controls (*p* = 0.048, Fig. 5b), which confirms that the active animals were less anxious. These results were consistent with the OF test, in which the running mice, but not the inactive controls, explored more intensively the center compartment of the cage (*p* = 0.011, Fig. 5c). In addition, the running groups spent approximately the same time in the center as compared to cage margins. In contrast, the inactive mice spent less time in the center area and more in the margin (*p* = 0.002, Fig. 5d). Additional functional parameters of the OF test are provided in Table 3.

**Table 3:** Functional parameters measured by the open field test. Results are presented as median (min-max)

	Inactive	Running	*P* value
No. of moves	90 (81–116)	88 (78–107)	0.69
Move time (s)	466 (361–491)	440 (346–486)	0.046
Velocity (cm/s)	5 (3.9–7)	4.6 (3.5–6.3)	0.24
Distance moved (cm)	2390 (1632–3343)	2048 (1210–2921)	0.13

## Discussion

Non-targeted approaches of metabolomic analysis allow for comprehensive understanding of the biology of exercise. The novelty of the present study is the determination of the cortical and the hippocampal metabolomic profiles in a mouse model of prolonged voluntary exercise, while previous investigations focused primarily on the measurements of composition of metabolites in various peripheral tissues of trained animals or in body fluids of humans [44]. Based on the obtained results, we propose that physical activity has a profound impact on the brain metabolome, which may contribute to the impact of exercise on cerebral homeostasis and functioning. Even more interesting, metabolomic responses to exercise were distinct in the hippocampus compared to the FC, indicating finely tuned responses from the individual brain regions.

Among the pool of significantly changed metabolites identified herein (16 in the hippocampus and 15 in the FC), only selected compounds were previously found to be impacted by exercise in human plasma [45] and in the peripheral tissues of non-human origin [46]. For example, plasma indicators of the TCA cycle (succinate, malate, and fumarate), lipolysis (glycerol), as well as modulators of insulin sensitivity (niacinamide) were changed in exercised volunteers [45]. Selected compounds distinguished by us in the hippocampus and the FC were also changed in the skeletal (fructose-6-phosphate, 2-aminoadipic acid, heptadecanoic acid, stearic acid and oleic acid) and cardiac (malic acid, creatinine) muscles of exercised rats [46].

A striking observation in the present study is that lipogenesis was increased in the hippocampi of the running mice. We observed that exercise induced expression of the hippocampal (but not cortical) FASN protein, the crucial enzyme of palmitate biosynthesis. This finding is consistent with a previous report that exercise upregulates FASN mRNA exclusively in the hippocampus of running mice [47]. However, it is important to notice that the levels of even-chain saturated FAs, including myristic acid (14:0), palmitic acid (16:0), and stearic acid (18:0), i.e., the primary end products of de novo lipogenesis [38], were elevated in both in the hippocampi and the FC. In order to explain this phenomenon, we propose that physical activity is a stressing factor which enforces systemic adaptation in the brain. During exercise, the brain requires a higher energy demand; therefore, it launches compensative mechanisms. We suspect that these mechanisms are turned on differentially during exercise and a resting period. The hippocampus is better equipped than the FC to use fats as energetic substrates via the TCA cycle, possessing the highest mitochondrial spare respiratory capacity among the brain structures [48]. The term ‘spare respiratory capacity’ or ‘reserve respiratory capacity’ is used to describe the amount of extra ATP that can be produced by oxidative phosphorylation in case of a sudden increase in energy demand [49]. Therefore, distinct metabolic adaptations are likely to be launched in the hippocampus and the FC. During the resting phase, lipogenesis is boosted in the hippocampus resulting in producing fats. These energetic substrates could then be utilized during the active phase, when hippocampal mitochondrial capacity is enhanced. In the FC different mechanisms may occur, because this brain region possess lower spare respiratory capacity [48], and mitochondrial efficiency cannot be elevated during exercise. During the resting phase, cortical TCA cycle may slow down, allowing for accumulation of FAs, which can be deposited as a fuel for the upcoming exercise. It should be noticed that the expression of FASN was higher in the hippocampi as compared to the FC. These differences could be influenced by a distinct cellular composition of these brain structures. Specifically, the hippocampi are more enriched with astrocytes than the FC [50], and it was shown that FASN is expressed by glial cells but not by neurons [51]. Physical activity can also increase the number of astrocytes [52] and induce their biochemical reprograming [53]. Thus, astrocytes are likely to contribute to metabolic adaptation to energetic stress and distinct responses induced in the hippocampi and the FC during exercise as observed in our study.

We next hypothesized that increased accumulation of FAs in the brain of exercised mice may have important functional outcomes. Indeed, the identified mixture of accumulated even-chain saturated FAs was previously recognized as causing anxiolytic-like effects when administered to rodents [43, 54]. These FAs have been shown to be consistently present in human amniotic fluid and, when administered to rats, they exerted a potent anxiolytic-like impact as manifested by increased burying latency, reduced cumulative burying, and increased time spent in the open arms of the elevated plus maze. These effects were observed in rodents of both sexes, did not depend on sex hormones, and were not associated with altered general locomotor activity [54]. Among the saturated FAs, exposure to myristic acid appeared to be primarily responsible for the anxiolytic-like effects [43]. Thus, it was a surprising finding that a similar anxiolytic-like impact can be achieved by exercise by modification of the FA profile in the brain. This observation was confirmed in a battery of three behavioral tests (namely, EPM, D/L, and OF tests) that are commonly used to measure anxiety level. Running mice demonstrated anxiolytic phenotype in these tests, the finding that is supported by several literature reports [8, 55].

The anxiolytic effect of FAs is well recognized for unsaturated omega-3 s [56]. In contrast, we have correlated the accumulation of saturated FAs with decreased anxious behavior as a consequence of exercise. Only a few studies so far examined the brain lipidomic composition during exercise. In the study of Chorna et al. physical activity increased hippocampal levels of palmitic and stearic acids in 20-weeks old C57BL/6 J mice, which is consistent with our findings [47]. On the other hand, research of Santos-Soto et al. reported exercise-related increases in cortical docosahexaenoic and arachidonic acids in younger mice, which was connected to anxiolytic effect [57]. These different effects may suggest that exercise impacts brain lipidome depending on the age of animals [47, 57]. However, the question of whether physical activity can influence behavior in a FA-dependent manner remains open and requires more detailed examination.

It should be emphasized that exercise acts systemically and its behavioral outcomes likely involve a wide range of effects from systemic to intracellular level. Among the hierarchized intracellular processes, the kynurenine pathway, which utilizes tryptophan, plays an important role in regulation of anxiety and mood disorders [58]. Picolinic acid is one of the metabolites produced in this pathway; therefore, it was interesting that its levels were elevated in the hippocampus of the running mice, pointing to an increase in turnover of the kynurenine pathway without tryptophan depletion. Indeed, the levels of tryptophan were unchanged between the running and inactive mice. Picolinic acid has low permeability through the blood brain barrier [59]; thus, the observed changes in its levels likely resulted from increased synthesis in the brain and did not originate in the periphery. There are a number of biological factors that can potentially affect picolinic acid levels and its brain synthesis, including age, circadian rhythms, and hormonal or nutritional factors [59]. The importance of picolinic acid stems from the fact that it may play a role in the regulation of glucose/energy metabolism [59], suggesting its involvement in the regulation of hippocampal metabolism during exercise.

## Conclusions

The voluntary wheel running differently modulated the hippocampal and cortical composition of metabolites ensuring higher cellular energetic demands. Increased physical activity was associated with upregulation of hippocampal lipogenesis and highly specific alterations in FA composition. Simultaneously exercise resulted in less anxious behavior. Altogether, our study links physical activity to highly specific changes in brain metabolite profile that can affect behavioral modifications.

## Supplementary information

**Additional file 1 **: **Figure 1.** Representative actinogram of a wheel running mouse. Active episodes are depicted as black dots during a 43 day running period (y axis). X axis presents the time of day/night cycle (12:12), with light phase lasting from 7 to 19, when dark phase began. Mice were active predominantly during dark phase of the cycle. **Table S1.** Metabolome profile of the hippocampus and frontal cortex of the running and inactive mice (*n* = 6–7). Data are presented as mean +/− S.D. (student T-test) or median with min. To max. (U Mann-Whitney test; marked by italics).

## Data Availability

The datasets analyzed during the current study available from the corresponding author upon request.

## Abbreviations

BrdU: Bromodeoxyuridine
DCX: Doublecortin
D/L: Dark/light test
EPM test: Elevated plus maze test
FA: Fatty acids
FASN: Fatty acid synthase
FC: Frontal cortex
OF test: Open field test
TCA: Tricarboxylic acid
