# Supplementary material for: Physical activity reduces anxiety and regulates brain fatty acid synthesis
Source: Mol Brain. 2020 Apr 17;13:62. doi: 10.1186/s13041-020-00592-7 (PMC7165435; doi:10.1186/s13041-020-00592-7)
Supplement: Supplementary file 1 — Additional file 1 : Figure 1. Representative actinogram of a wheel running mouse. Active episodes are depicted as black dots during a 43 day running period (y axis). X axis presents the time of day/night cycle (12:12), with light phase lasting from 7 to 19, when dark phase began. Mice were active predominantly during dark phase of the cycle. Table S1. Metabolome profile of the hippocampus and frontal cortex of the running and inactive mice (n = 6–7). Data are presented as mean +/− S.D. (student T-test) or median with min. To max. (U Mann-Whitney test; marked by italics). [file 13041_2020_592_MOESM1_ESM.docx]

**Exercise Regulates Fatty Acid Synthesis In The Mouse Brain And Promotes Anxiolytic Effect**

**Arkadiusz Liśkiewicz^1,2^, Marta Przybyła^1,3^, Anna Wojakowska^4^, Łukasz Marczak^4^, Katarzyna Bogus^5^, Marta Nowacka-Chmielewska^1,3^, Daniela Liśkiewicz^1,3^, Andrzej Małecki^1^, Jarosław Barski^3^, Joanna Lewin-Kowalik^2^, Michal Toborek^1,6^**

**^1^**Laboratory of Molecular Biology, Faculty of Physiotherapy, The Jerzy Kukuczka Academy of Physical Education, Katowice 40-065, Poland

**^2^**Department of Physiology, Medical University of Silesia, Katowice 40-752, Poland

**^3^**Department for Experimental Medicine, Medical University of Silesia, Katowice 40-752, Poland

**^4^**Institute of Bioorganic Chemistry, Polish Academy of Sciences, Poznan 61-704, Poland

**^5^**Department of Histology, School of Medicine in Katowice, Medical University of Silesia, Katowice 40-752, Poland

**^6^**Department of Biochemistry and Molecular Biology, University of Miami School of Medicine, 1011 NW 15th Street, Miami, FL, 33136, USA

Supplementary information contains:

Supplementary Fig. 1. Actinogram of running activity

Supplementary Table 1. List of significantly different compounds discriminated as the result of metabolomic profiling


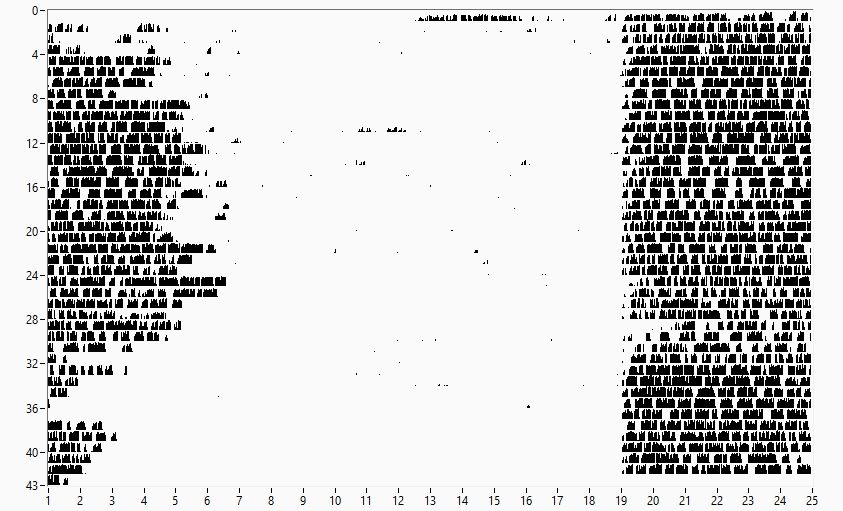


Supplementary Figure 1. Representative actinogram of a wheel running mouse. Active episodes are depicted as black dots during a 43 day running period (y axis). X axis presents the time of day/night cycle (12:12), with light phase lasting from 7 to 19, when dark phase began. Mice were active predominantly during dark phase of the cycle.

Table 1. Metabolome profile of the hippocampus and frontal cortex of the running and inactive mice (n=6-7). Data are presented as mean +/- S.D. (Student T-test) or median with min. to max. (U Mann-Whitney test; marked by italics).

|  | **Hippocampus** | | | | **Frontal cortex** | | | | **Replib, Mainlib, Fiehn library identification** |
| --- | --- | --- | --- | --- | --- | --- | --- | --- | --- |
| **Compound** | **Inactve** | **Runnners** | **P value** | **Runnners as % of inactive** | **Inactve** | **Runnners** | **P value** | **Runnners as % of inactive** |  |
| **Compounds significantly changed in hippocampus** | | | | | | | | | |
| **Creatinine** | 19216026 (±10617256) | 8509740 (±4709227) | 0.048 | 44 | 7265821 (±4589836) | 12908408 (±8876126) | 0.16 | 178 | 1-Methyl-N,N-bis(trimethylsilyl)-4-[(trimethylsilyl)oxy]-1H-imidazol-2-amine |
| **Gluconic acid** | 374548 (±71522) | 239732 (±36544) | 0.0021 | 64 | 322179 (±41093 | 361408 (±111339 | 0.4 | 112 | D-Gluconic acid, 2,3,4,5,6-pentakis-O-(trimethylsilyl)-, trimethylsilyl ester |
| **Uridine 5'-monophosphate** | 2894007 (±379676) | 2155021 (±289947) | 0.004 | 74 | 2343224 (±410887) | 2575199 (±563463) | 0.4 | 110 | 2(1H)-Pyrimidinone, 1-[2,3-bis-O-(trimethylsilyl)-?-D-ribofuranosyl]-4-(trimethylsiloxy)-, 5'-[bis(trimethylsilyl) phosphate] |
| **Picolinic acid** | 62813 (±40081) | 148910 (±53144) | 0.0100 | 237 | 142709 (±65263) | 114137 (±99952) | 0.54 | 80 | Picolinic acid, trimethylsilyl ester |
| **L-Valine** | 5049080 (±582330) | 4286543 (±371746) | 0.022 | *85* | *4504077 (2818898-4804983)* | *4445456 (3149737-4533205)* | *0.46* | 99 | L-Valine, N-(trimethylsilyl)-, trimethylsilyl ester |
| **Acetylated lysine** | *1969639 (198297-2246426)* | *2288958 (1950077-2595534)* | *0.0367* | 116 | 1406630 (±268706) | 1526029 (±190991) | 0.36 | 108 | N-α-Acetyl-L-Lysine, N,N-trimethylsilyl-, trimethylsilyl ester |
| **Sedoheptulose 7-phosphate** | 38833 (±5240) | 53417 (±13025) | 0.0291 | 138 | 43971 (±17000) | 45244 (±11448) | 0.87 | 103 | D-Altro-2-Heptulose, 1,3,4,5,6-pentakis-O-(trimethylsilyl)-, O-methyloxime, 7-[bis(trimethylsilyl) phosphate] |
| **4-Ketoglucose** | 79685 (±22996) | 113598 (±20058) | 0.017 | 143 | 62214 (±16637) | 61060 (±22534) | 0.92 | 98 | 4-Ketoglucose, bis(O-methyloxime), tetrakis(trimethylsilyl) |
| **Urea** | 40006466 (±15247368) | 57624242 (±7687600) | 0.03 | 144 | 46106245 (±19065836) | 48916564 (±21227194) | 0.8 | 106 | Urea, N,N'-bis(trimethylsilyl)- |
| **Fructose 6-phosphate** | *142273 (52400-207948)* | *231833 (173510-539473)* | *0.026* | 163 | 168477 (±135215) | 153036 (±50082) | 0.78 | 91 | d-Fructose, 1,3,4,5-tetrakis-O-(trimethylsilyl)-, o-methyloxime, 6-[bis(trimethylsilyl) phosphate] |
| **3-Phosphoglycerate** | 587120 (±180032) | 1034352 (±328683) | 0.0152 | 176 | 514515 (±241765) | 437913 (±127916) | 0.47 | 85 | Propanoic acid, 3-[[bis[(trimethylsilyl)oxy]phosphinyl]oxy]-2-[(trimethylsilyl)oxy]-, trimethylsilyl ester, (S)- |
| **Galactose-6-phosphate** | 103105 (±26195) | 198350 (±73226) | 0.0133 | 192 | 123095 (±95459) | 108068 (±25039) | 0.69 | 88 | d-Galactose, 2,3,4,5-tetrakis-O-(trimethylsilyl)-, O-methyloxime, 6-[bis(trimethylsilyl) phosphate] |
| **D-Pinitol** | 19037 (±9074) | 82033 (±8408) | <0.0001 | *431* | *37701 (17100-75501)* | *49999 (26500-179896)* | *0.38* | 133 | D-Pinitol, pentakis(trimethylsilyl) ether |
| **Compounds significantly changed in frontal cortex** | | | | | | | | | |
| **L-Arabitol** | 262723 (±48223) | 210468 (±37727) | 0.05 | 80 | 359331 (±68652) | 242379 (±41575) | 0.0023 | 67 | L-(-)-Arabitol, pentakis(trimethylsilyl) ether |
| **Glycerol** | *11531 (3521-3384779)* | *2172227 (1913749-2677249)* | *0.31* | *18838* | *2371716 (1274323-3190387)* | *1634706 (6576-2449235)* | *0.038* | 69 | Glycerol, tris(trimethylsilyl) ether |
| **D-Ribose** | 42929 (±21655) | 47718 (±6374) | 0.61 | 111 | 53286 (±7715) | 38814 (±12994) | 0.026 | 73 | d-Ribose, 2,3,4,5-tetrakis-O-(trimethylsilyl)-, O-methyloxime |
| **Aminoadipic acid** | 236170 (±54153) | 182550 (±43884) | 0.08 | 77 | 388013 (±87431) | 284100 (±70208) | 0.03 | 73 | α-Aminoadipic acid, triTMS |
| **Niacinamide** | 4040615 (±1648891) | 3558015 (±571339) | 0.51 | 88 | 2184893 (±463775) | 1585933 (±453801) | 0.031 | 73 | Niacinamide, N-trimethylsilyl- |
| **Phosphoetanoloamine** | 15854767 (±6799777) | 18982645 (±1870929) | 0.3 | 120 | 22555158 (±2736147) | 17161754 (±5569745) | 0.04 | 76 | Phosphoric acid, 2-[bis(trimethylsilyl)amino]ethyl bis(trimethylsilyl) ester |
| **Uridine** | 782298 (±259524) | 797386 (±115792) | 0.8981 | 102 | 808964 (±191024) | 617083 (±121548) | 0.045 | 76 | Uridine, 2',3',5'-tris-O-(trimethylsilyl)- |
| **alpha-Ketoglutaric acid** | 26960632 (±5447839) | 30972231 (±4172623) | 0.17 | 115 | 21201955 (±2826245) | 16574158 (±2867022) | 0.014 | 78 | 2-Ketoglutaric acid ditms |
| **Fumaric acid** | *2186058 (1473790-5652666)* | *2046656 (1631311-2123629)* | *0.45* | *94* | *2117793 (1982996-* *3344989)* | *1747135 (1425859-* *2057028)* | *0.0083* | 82 | 2-Butenedioic acid (E)-, bis(trimethylsilyl) ester |
| **Citric acid** | 5447019 (±1334094) | 5442578 (±682500) | 0.99 | 100 | 6769390 (±728139) | 5535689 (±1158222) | 0.04 | 82 | 1,2,3-Propanetricarboxylic acid, 2-[(trimethylsilyl)oxy]-, tris(trimethylsilyl) ester |
| **Phenylalanine** | 1352836 (±288170) | 1346592 (±209568) | 0.96 | 100 | 1225972 (±90679) | 1018852 (±193422) | 0.025 | 83 | N,O-Bis-(trimethylsilyl)phenylalanine |
| **Butane, 1,2,3,4-tetrakis[(trimethylsilyl)oxy]-** | 144539 (2491-166897) | 144982 (122949-172939) | 0.63 | 100 | 134309 (116567-209865) | 112417 (4084-130655) | 0.026 | 84 | 3,8-Dioxa-2,9-disiladecane, 2,2,9,9-tetramethyl-5,6-bis[(trimethylsilyl)oxy]-, (R*,S*)- |
| **Malic acid** | 3502978 (±955127) | 3834061 (±783187) | 0.51 | 109 | 4301218 (±455259) | 3725586 (±505600) | 0.044 | 87 | Butanedioic acid, [(trimethylsilyl)oxy]-, bis(trimethylsilyl) ester |
| **gamma-Aminobutyric acid (GABA)** | 114044024 (±16202564) | 111500000 (±8803408) | 0.74 | *98* | *97189694 (91813311-113490791)* | *84872862 (12608558-93502396)* | *0.0023* | 87 | Butanoic acid, 4-[bis(trimethylsilyl)amino]-, trimethylsilyl ester |
| **Pelargonic acid** | 116275 (±17167) | 101812 (±18170) | 0.17 | 88 | 108335 (±22876) | 141788 (±31963) | 0.044 | 131 | Nonanoic acid, trimethylsilyl ester |
| **Compounds significantly changed in hippocampus and frontal cortex** | | | | | | | | | |
| **Methyl arachidonoic acid** | *107115 (86499-496867)* | *79850 (52499-131117)* | *0.035* | 75 | 85192 (±59230) | 26518 (±20591) | 0.029 | 31 | 5,8,11,14-Eicosatetraenoic acid, methyl ester, (all-Z)- |
